# Spatial distribution and associated factors of co-occurrence of overweight/obesity and Anemia among women in the reproductive age in sub-Saharan Africa: A multilevel analysis, DHS 2016–2021

**DOI:** 10.1371/journal.pone.0299519

**Published:** 2024-04-18

**Authors:** Mihret Getnet, Wullo Sisay Sisay, Adugnaw Zeleke Alem

**Affiliations:** 1 Department of Human Physiology, School of Medicine, College of Medicine and Health Sciences, University of Gondar, Gondar, Ethiopia; 2 Department of Epidemiology and Biostatistics, Institute of Public Health, College of Medicine and Health Sciences, University of Gondar, Gondar, Ethiopia; University for Development Studies, GHANA

## Abstract

**Background:**

Overweight/obesity is one of the major public health problems that affect both developed and developing nations. The co-occurrence of overweight/obesity and anemia is thought to be largely preventable if detected early. To date, no spatial analyses have been performed to identify areas of hotspots for the co-occurrence of overweight/obesity and anemia among reproductive women in sub-Saharan Africa. Therefore, this study aimed to assess the spatial distribution and associated factors of the co-occurrence of overweight/obesity and anemia among women of reproductive age.

**Methods:**

Data for the study were drawn from the Demographic and Health Survey, a nationally representative cross-sectional survey conducted in the era of Sustainable Development Goals, in which the World Health Assembly decided and planned to cease all forms of malnutrition by 2030. Seventeen sub-Saharan African countries and a total weighted sample of 108,161 reproductive women (15–49 years) were included in our study. The data extraction, recoding and analysis were done using STATA V.17. For the spatial analysis (autocorrelation, hot-spot and interpolation), ArcGIS version 10.7 software, and for the SaTScan analysis, SaTScan version 10.1 software was used. Descriptive statistics were presented using frequency tables and percentages. We employed multilevel logistic regression to investigate associated factors. In the multivariable analysis, variables with a p-value of ≤0.05 are considered as a significant factor associated with co-occurrence of overweight/obesity and anemia among women aged 15–49 years.

**Results:**

The overall co-occurrence of overweight/obesity and anemia among women in sub-Saharan Africa was 12% (95%CI: 9–14%). The spatial analysis revealed that the co-occurrence of overweight/obesity and anemia among women significantly varied across sub-Saharan Africa. (Global Moran’s I = 0.583163, p<0.001). In the spatial window, the primary-cluster was located in Liberia, Guinea, Gambia, Sira Leon, Mauritania, Mali, Cameron and Nigeria with a Log-Likelihood Ratio (LRR) of 1687.30, and Relative Risk (RR) of 2.58 at a p-value < 0.001. In multilevel analysis, women aged 25–34 years (AOR = 1.91, 95%CI: 1.78, 2.04), women aged 35–49 years (AOR = 2.96, 95% CI: 2.76, 3.17), married (AOR = 1.36, 95% CI: 1.27, 1.46), widowed (AOR = 1.22, 95%CI: 1.06, 1.40), divorced (AOR = 1.36, 95% CI: 1.23, 1.50), media exposure (AOR = 1.31, 95%CI: 1.23, 1.39), middle income (AOR = 1.19, 95%CI: 1.11, 1.28), high income/rich (AOR = 1.36, 95%CI: 1.26, 1.46), not working (AOR = 1.13, 95% CI:1.07, 1.19), traditional contraceptive utilization (AOR = 1.39, 95%CI: 1.23, 1.58) and no contraceptive use (AOR = 1.27, 95%CI: 1.20, 1.56), and no health insurance coverage (AOR = 1.36, 95%CI: 1.25, 1.49), were individual level significant variables. From community-level variables urban residence (AOR = 1.61, 95%CI: 1.50, 1.73), lower middle-income country (AOR = 2.50, 95%CI: 2.34, 2.66) and upper middle-income country (AOR = 2.87, 95%CI: 2.47, 3.34), were significantly associated with higher odds of co-occurrence of overweight/ obesity and anemia.

**Conclusion and recommendations:**

The spatial distribution of the co-occurrence of overweight/obesity and anemia was significantly varied across the sub-Saharan African country. Both individual and community-level factors were significantly associated with the co-occurrence of overweight/obesity and anemia. Therefore, public health programmers and other stalk holders who are involved in maternal healthcare should work together and give priority to hotspot areas of co-occurrence in sub-Saharan Africa.

## Introduction

### Statement of the problem

Reproductive women who enter pregnancy with the co-occurrence of overweight/obesity and anemia confront adverse maternal, obstetric and birth outcomes linked to both anemia and adiposity [1]. The simultaneous occurrence of overweight/obesity and anemia, is mostly caused by dilutional hypoferremia, poor dietary iron intake, increased iron requirements, and/or impaired iron absorption with an increase in obesity [2].

Anemia is a global health problem, characterized by a reduction in the number of red blood cells (RBCs) and/or hemoglobin (Hb) concentration, which affects the ability of blood to carry oxygen for physiologic requirements [3]. Non-pregnant women (NPW), are considered as anemic when their Hb concentration is < 11.9 g/dL [4,5] and it affects about 30% nonpregnant women globally [6].

Similarly, overweight/ obesity (OWOB) is one of the major public health burden of developed and developing nations [7,8]. Overweight and/or obese have been identified by the World Health Organization (WHO) as the fifth potential cause of death worldwide [9] and its magnitude is expected to increase without stopping measure [10]. Surprisingly, the co-occurrence of overweight/obesity and anemia increases annually in different WHO regions by 0.47 percentage points in Southeast Asia, 0.24 percentage points in West and Central Africa [11].

Being overweight/ obese and anemic has a catastrophic effect on women since it affects not only their health but also the health of their child [12,13]. The co-occurrence of the problem is rising quickly in developing nations, as a result of the growth of their economies, a decline in physical activity, and a shift in the diet that favors high energy and fat intake [14]. Its prevalence among reproductive age group women was 33.6% in Maldives [13], 15.3% in Pakistan and 18.6% in Afghanistan [15], and 6.7% in Ghana [16].

Regarding factors associated with co-occurrence of OWOB and anemia: being older age, married and richer wealth status [16,17], urban residency [16,18], being separated/divorced, widowed, unemployed, level of educational attainment [17], were significantly associated with co-occurrence of overweight/obesity and anemia. Besides, television watching [19,20], higher education [21], not working [19], use of hormonal contraception [22,23], and type of toilet facility [24], were associated with overweight and obesity. These factors lead to the development of anemia but more often coexist, which can be an indicator of both nutrition and health status [25]. Moreover, NPWs of childbearing age are more likely to suffer from anemia due to lack of nutritional intake [26], and heavy bleeding that may last longer than five days [27,28].

Even though the magnitude of co-occurrence of overweight/obesity and anemia among reproductive women is a common problem in sub-Saharan African countries, currently, there are limited studies conducted to identify the prevalence and determinant factors in this population group (NPW) at the national level [16,18]. Concomitantly, more than 50% of anemic cases during pregnancy begin at the date of conception [29]. Premature delivery, low birth weight, and fetal and maternal mortality have all been linked to the beginning of pregnancy with depleted iron stores and/or low Hb concentration [26].

The simultaneous occurrence of overweight/ obesity and Anemia was thought to be largely preventable, easy to treat if detected early, and approaches of control and prevention well-documented [17,18]. In 2012, the 65th World Health Assembly approved an action plan and global targets for maternal, infant, and child nutrition, with a target to reduce anemia prevalence by 50% and zero growth of overweight/obesity in reproductive-age women by 2025, standing from 2011 levels [30,31]. Sustainable Development Goals additionally plan to cease all forms of malnutrition by 2030 [32].

Despite these facts, the overall progress has been insufficient and still, it has continued to be a common cause of mortality and morbidity [16]. Suggested strategies aimed at prevention of co-occurrence of overweight/ obesity and anemia are focused on the major underlying causes and their risk factors [16,17]. However, there is a lack of data on the co-occurrence of OWOB and anemia, and geographically variable factors in developing nations.

To the best of our searching, no spatial analyses have been performed to identify areas of hotspots (high prevalence) of co-occurrence of overweight/ obesity and anemia among reproductive women in SSA. Assessing the geographic distributions of overweight/ obesity and anemia co-occurrence, and its associated factors by area is important for prioritizing target prevention and intervention programs among reproductive women.

Therefore, this study aimed to assess the spatial distribution and associated factors of co-occurrence of overweight/ obesity and anemia among reproductive-age women in Sub-Saharan Africa.

## Methods and materials

### Study design and setting

The current study was based on demographic and health surveys conducted in sub-Saharan Africa (SSA). A cross-sectional study design was employed based on nationally representative Demographic and Health Survey (DHS) data between 2016 and 2021 in Sub-Saharan African countries.

### Data sources

Since 1984, the DHS has been conducted in more than 85 countries globally. The DHS collects a wide range of objective and self-reported data with strong evidence on indicators such as adult self-reported health behaviors, mortality, nutrition, and measures of fertility, reproductive health, mother and child health, and mortality. The main advantage of DHS is its high response rates, national coverage and standardized data collection procedures across countries and consistent content over time. Data from DHS encourages epidemiological research focused on monitoring prevalence, trends and inequalities. It takes nationally representative samples of the country’s population. It was collected using a structured, interviewer-administered questionnaire every five years. The present study used DHS and was conducted after sustainable development goal endorsement (2016–2021) in sub-Saharan countries.

### Populations

#### Source population

All reproductive women aged 15–49 years across SSA countries

#### Study population

All reproductive women aged 15–49 years in 19 SSA countries during data collection

### Inclusion criteria

All reproductive women aged 15–49 years who were in selected EAs of SSA countries and whose data recorded in the standard DHS data set was included.

### Exclusion criteria

Women who were pregnant, underweight, had incomplete observation/ record (either BMI or Hb) in the DHS data set. Two countries, namely Angola and Zambia were excluded from the study because of lack of recorded of BMI and Hb.

### Sample size and sampling procedure

**Fig 1:**
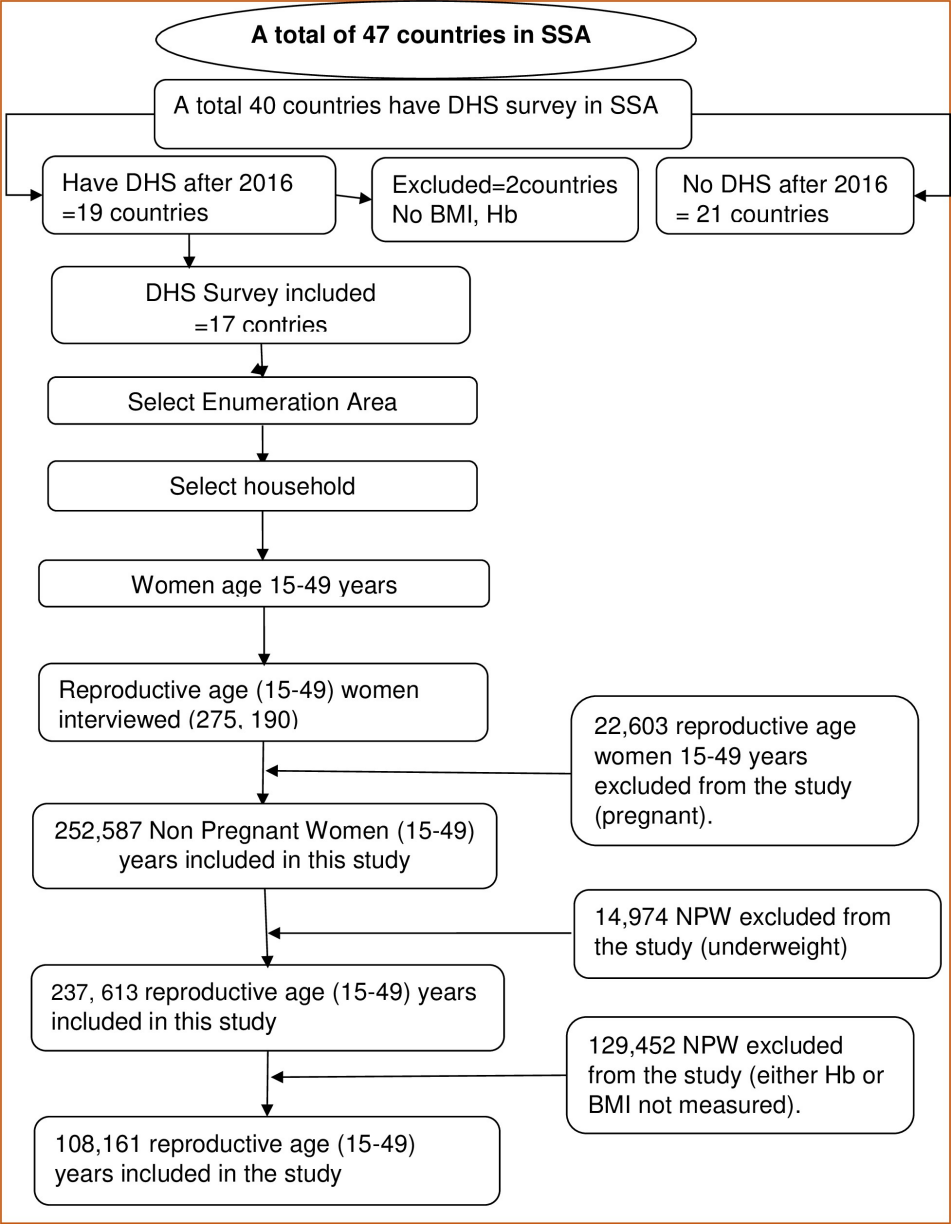
Flow diagram showing the sampling procedure of DHS data.

In the present study, a total of 108,161 women of reproductive age group were included (Fig 1). The DHS follows a multistage stratified random sampling technique. A two-stage stratified cluster sampling was used. In the first stage, the number of households needed per geographical area is determined, and clusters (or census enumeration areas) are randomly selected with a probability proportional to size. The second stage consists of a random selection of households within the selected clusters. For each sampled household, standard model questionnaires are then employed to collect primary data at the household and individual levels. Therefore, a total of 594 sampling strata have been created. Then, each stratum was again further divided into enumeration areas (EAs; a geographic area consisting of 200–300 households, which served as a counting unit for the census), using the list of all EAs (clusters). In the first stage, a total of 10,859 EAs were selected. The EAs were selected with a probability proportional to the EA size and with independent selection in each sampling stratum. In the second stage, a fixed number of households per cluster were selected randomly from the household listing. A total of 275,190 women (15–49 years) were interviewed, making up response rates of 91.8% (Table 1).

**Table 1:** Study participants interviewed by country and their respective year of survey.

Country	Year of survey	Frequency	Percent (%)
Benin	2017/ 18	14,228	5.6
Burundi	2016/ 17	15,921	6.3
Cameron	2018	12,338	4.9
Ethiopia	2016	14,561	5.8
Gambia	2019/ 20	10,890	4.3
Guinea	2018	9,928	3.9
Liberia	2019/ 20	7,442	2.9
Madagascar	2021	17,584	7.0
Mali	2018	9,374	3.7
Mauritania	2019/ 21	14,614	5.8
Malawi	2015/ 16	22,729	9.0
Nigeria	2018	37,630	15.0
Rwanda	2019/ 20	13,791	5.4
Sierra Leone	2019	14,584	5.8
Tanzania	2015/ 16	12,129	4.8
Uganda	2016	16,634	6.6
South Africa	2016	8,210	3.2
Total		252,587	100

Therefore, the total sample size from the pooled data to be analyzed in this study was 108,161, after excluding those women with no hemoglobin and BMI recorded and those who were underweight. The total number of nonpregnant women from each country ranged from 8,210 in South Africa to 37,630 in Nigeria with their respective years of survey (Table 1).

### Study variables

#### Outcome variable

The outcome variable for this study is the Co-occurrence of OWOB and anemia. If it fulfills the occurrence of anemia in either level (mild, moderate or severe) with overweight/ obesity, and it was dichotomize as yes/no. ‘Yes’ indicates there is a simultaneous occurrence of overweight/obesity and anemia. But if there was an occurrence of anemia without overweight/obesity and the reverse, it was considered as ‘No’.

#### Independent variables

For this study both individual and community level variables were considered.

**The individual and household level factors were:** age, marital status (labeled as single, married, divorced, separated and widowed), maternal occupation (working or not working), maternal education (no education, primary education, secondary education, higher education), media exposure (reading magazine, listening radio, watching television, using internet), sex of household head, smoking, health insurance coverage, wealth index (poorest, poorer, middle, richer and richest), contraceptive use (no, use traditional method, use modern method), perception of distance from the health facility, source of drinking water and type of toilet facility.

**Community level variables:** place of residence (urban or rural), sub-Saharan regions (eastern, western, central and southern), and country income status were considered.

### Operational definitions

**Body mass index** (BMI): was dichotomized as normal and overweight/ obesity. Individuals are found to have a normal BMI when their BMI is between 18.5 to 24.9kg/m^2^. Those individuals with a BMI of ≥ 25kg/m^2^ were overweight and those with BMI ≥ 30kg/m^2^ were obese [16]. According to DHS data height and weight, measurements were carried out on women aged 15–49 years in all selected households. Weight measurements were taken by using portable SECA mother-infant scales with a digital display that were developed and produced with the guidance of UNICEF. Height measurements were carried out using a Shorr measuring board.

**Anemia status**: based on the DHS guideline, Non-pregnant women with a hemoglobin level equal to or below 11.9 g/dl were considered anemic. On the other hand, mild anemia, defined by hemoglobin levels between 10.0–11.9 g/dl, moderate anemia: hemoglobin level between 7.0–9.9 g/dl, and severe anemia: hemoglobin level less than 7.0 g/dl in non-pregnant women, respectively [33]. We consider these mild, moderate and severe level of anemia as “anemic” and otherwise “non-anemic”.

**Media exposure**: Exposure to either media was assessed by recoding watching television ‘yes/ no’, listening radio ‘yes/ no’, reading newspaper ‘yes/ no’ and internet use ‘yes/ no’. We sum these four means of media access to generate exposure to media and dichotomized into yes, if women use at least one of either watching television, listening radio, reading newspaper, or internet, and no, if women have no media exposure [34].

**Toilet facility**: **Improved** (private facility with/without septic tank) or **unimproved** (shared/ public latrine, pit latrine without slab, yard/bush/ forest, river/stream/creek, and others) [35].

**Drinking water sources**: **protected** (piped water, well with pump, protected well, protected spring, and rain) or **unprotected sources** (unprotected well, unprotected spring, bottled water, refilled water, river/ lake/pond/irrigation/dam, and other) [36].

**Country wealth status:** The country’s wealth status was categorized as low income, lower middle income, and upper middle-income country based on the World Bank List of Economies classification since 2022 [37].

### Data quality control

The DHS data across all nations were comparable. The DHS guideline provided a precise definition of the missing values. Since the DHS survey is a cross-sectional study, the variables were removed from further analysis if the missing value in the explanatory variables was greater than 5%. To ensure the quality of the data, the data extractions were carried out by public health professionals who have experience with DHS data. The Individual record (IR) file was cleaned up before the data were extracted using standard DHS procedures. Data consistency for each of the 19 countries has been checked before being appended.

Standardized interview questions were used to measure each variable uniformly in the DHS. In order to compile conveniently, they recoded uniformly with other countries during data management. The primary investigator and supervisor have assessed the data daily for consistency and completeness. The extent of co-occurrence of overweight/obesity and anemia among women in each nation was assessed with the respective DHS reports.

### Data management and statistical analysis

The data were checked for completeness and weighted before doing any statistical analysis. The data extraction, recoding and analysis were done using STATA V.17. A sampling weight was done to adjust for the non-proportional allocation of the sample to different regions and the possible differences in response rates. Hence, the actual representativeness of the survey results at both the national and regional levels was ensured.

### Spatial analysis

For the spatial analysis ArcGIS version 10.7software and for SaTScan analysis SaTScan version 10.1 software was used. Spatial auto correlation, interpolation, the hotspot and SaTScan analysis were performed under spatial analysis.

### Spatial autocorrelation analysis

The spatial autocorrelation (Global Moran’s I) statistic was used to measures whether co-occurrence of OWOB with anemia were dispersed, clustered or randomly distributed in the study area [38]. Moran’s I is a spatial statistic used to measure spatial autocorrelation by taking the entire data set and produce a single output value which ranges from -1 to +1. **Moran’s, I** Value close to −1 indicate co-occurrence of OWOB and anemia dispersed, whereas Moran’s I close to +1 indicate co-occurrence of OBOW and anemia clustered and co-occurrence distributed randomly if **I** value is zero. A statistically significant Moran’s I (p < 0.05) lead to rejection of the null hypothesis.

### Incremental spatial autocorrelation

Incremental spatial autocorrelation was done to obtain the maximum peak distance where co-occurrence clustering is more pronounced. The maximum peak distance is the distance where maximum spatial autocorrelation occurs and this was used as a distance band for hotspot analysis.

### Hot spot analysis (Getis-OrdGi* statistic)

Getis-OrdGi* statistics was performed to measure how spatial autocorrelation varies over the study location by calculating GI* statistic for each area. Z-score was computed to determine the significant hotspot and significant cold spot areas of co-occurrence of OWOB and anemia. From the statistical output with high GI* was used to indicate “hot-spot” whereas low GI* indicates “cold-spot” per proportion of co-occurrence of OWOB and anemia among reproductive women.

### Spatial interpolation

The Kriging spatial interpolation technique was used to predict the percentage of co-occurrence of OWOB and anemia among reproductive-age women on the un-sampled areas in the countries based on observed measurements. There are various deterministic and geostatistical interpolation methods. Among these, the ordinary Kriging spatial interpolation method was used for predictions of the co-occurrence of OWOB and anemia among women in unobserved areas of sub-Saharan Africa.

### Spatial scan (Sat scan) statistical analysis

In the spatial scan statistical analysis, a Bernoulli-based model was employed to identify statistically significant spatial clusters of reproductive women with co-occurrence of OWOB and anemia using Kuldorff’s SaTScan version 10.1software. Women with co-occurrence were taken as cases and those without co-occurrence were considered as controls to fit the Bernoulli model. The numbers of cases in each location had a Bernoulli distribution, and the model required data for cases, controls and geographic coordinates. A Likelihood Ratio (LR) test statistic and the p-value were applied to figure out whether the number of observed women who have co-occurrence of OWOB and anemia within the potential cluster was significantly higher than expected or not. The scanning window with the highest likelihood was the cluster that is most likely performing well (primary) cluster. For each identified cluster, the log-likelihood ratio (LLR) test statistic with its p- value, the relative risk (RR), the location radius, population, and cases were reported.

### Statistical analysis

The distributions of participants’ characteristics were examined using frequencies and percentages. The pooled co-occurrence of overweight/ obesity and anemia with the 95% Confidence Interval (CI) was reported using a forest plot.

### Multi-level modeling approach

We fitted a multilevel mixed-effects binary logistic regression model, since the standard binary logistic regression model’s independent assumption is violated by the hierarchal nature of DHS data. To choose the appropriate model for the study, four models were fitted. The first is the null model (Model I) which contains no exposure variables and used to check the variability of co-occurrence of OWOB and anemia in the community. Then Model II and Model III multilevel models were contained individual level variables and community level variables respectively. In the last model (Model IV), both individual and community level variables were fitted simultaneously with the outcome variable. Model comparison was done by using deviance and the model with the lowest deviance was selected as the best-fitted model. Both bivariable and multivariable multilevel logistic regression were performed to identify the determinants of co-occurrence of OWOB and anemia. Finally, to determine the associations of predictors, variables with a p-value of ≤0.2 at bivariable analysis were selected for multivariable analysis and in multivariable analysis, variables with a p-value less than 0.05 was considered as significantly associated factors.

### Parameter estimation method

**The fixed effects:** were being used to estimate the association between the likelihood of prevalence of co-occurrence of overweight/obesity and anemia and explanatory variables both at individual and community levels. Associations between dependent and independent variables were assessed and its strength has been presented using Adjusted Odds Ratio (AOR) and 95% confidence intervals with a p-value of < 0.05.


$$Log(\frac{\pi ij}{1-\pi ij})=\beta o+\beta 1xij+\beta 2xij+\ldots uj+eij$$

Where,

*πij*: the probability of co-occurrence of OWOB and anemia,

1 − *πij*: the probability of no co-occurrence of OWOB and anemia,

β1xij are individual and community level variables for the i^th^ individual in group j, respectively. The ß’s are fixed coefficients indicating a unit increase in X can cause a ß unit increase in probability co-occurrence of OWOB and anemia. While the ß_0_ is intercept that is the effect on co-occurrence of OWOB and anemia, when the effect of all explanatory variables are absent. The uj shows the random effect (effect of the community on the women co-occurrence of the problem) for the j^th^ community. The clustered data nature and the within and between community variations were taken into account assuming each community has a different intercept (β_0_) and fixed coefficient (β) [39,40].

**Random effects:** were used to estimate the mean distribution of effects, and to compare individuals from two different randomly chosen clusters. It was estimated by the median odds ratio (MOR), Intra Class Correlation Coefficient (ICC), and Proportional Change in Variance (PCV).

The MOR is defined as the median value of the odds ratio between the area at the highest risk and the area at the lowest risk when randomly picking out two clusters.

MOR  =  exp.[√(2 × VA) × 0.6745], or MOR = e^0.95√VA^ where; VA is the area level variance [41,42].

The PCV reveals the variation in the prevalence of co-occurrence of OWOB and anemia among women 15–49 years explained by factors. The PCV is calculated as; PCV = $\frac{Vnull-VA}{Vnull}$*100 where; V_null_  =  variance of the initial model, and VA  =  variance of the model with more terms.

The ICC which reveals the variation of co-occurrence of OWOB and anemia between clusters is calculated as; ICC = $\frac{VA}{VA+3.29}$*100%, where; VA  =  area/cluster level variance [42,43].

## Ethical consideration

We requested DHS Programme and permission was granted to download and use the data for this study from http://www.dhsprogram.com. Ethical clearance was taken from the Institutional Review Board of University of Gondar with reference number of IPH/ 2509/ 2023 to approve procedures for DHS public-use data sets. We did not in any way allow respondents, households or sample communities to be identified. Since there are no names of individuals or household addresses in the data files. The geographic identifiers only go down to the regional level (where regions are typically very large geographical areas encompassing several states/provinces). Each EA (primary sampling unit) has a number in the data file, but their numbers did not have any labels to indicate their names or locations. The data were collected after written informed consent had been obtained from the study participant and conducted according to the principles expressed in the declaration of **Helsinki**.

## Results

### Background characteristics of study participants

A total of weighted 108, 161 reproductive women aged 15–49 years were included in this study. More than half, 67,552 (62%) of women were above the age of 25 years, with a median age of 28 (IQR: 15) years. From the total of our study participants, nearly 70% of them had media exposure, and 69.6% of them have primary and above education.

The percentage of women concerning wealth status rich and middle income was 46.4% and 19.6%, respectively. Two-thirds of the women have an occupation (working).

From the community level factors (37.6%) of the study participants were from urban residences. In this study, sixty percent of SSA countries were under low -income countries (Table 2).

**Table 2:** Background characteristics of reproductive age women of sub-Saharan Africa demographic health survey, 2023.

Variables	Categories	Frequency		Weighted Percentage (%)
		Unweighted	weighted	
Age	15–24 years	41,164	41,172	37.9
	25–34 years	33,216	33,966	31.2
	35–49 years	33,781	33,586	30.9
Sex of household	Male	77,392	78,469	72.1
	Female	30,769	30,255	27.9
Marital status	Single	31,010	30,958	28.5
	Married	67,002	67,683	62.3
	Widowed	2,852	2, 869	2.6
	Divorced	7,297	7, 214	6.6
Educational attainment	No education	32,901	33,065	30.4
	Primary education	36,827	37,230	34.3
	Secondary & above	38,433	38,429	35.3
Media exposure	Yes	75,808	76, 023	69.9
	No	32,346	32,692	30.1
Wealth index	Poor	38,087	37, 028	34.0
	Middle	21,222	21, 256	19.6
	Rich	48,852	50, 440	46.4
Occupation	Working	69,385	70,451	66.8
	Not working	35,437	35,015	33.2
Source of water	Protected	78,575	78,039	71.8
	Unprotected	29,586	30,685	28.2
Type of toilet	Improved	55,175	55,350	50.9
	Unimproved	52,986	53,374	49.1
Contraceptive utilization	Modern method	26,743	27,825	25.6
	Traditional method	3,026	3,195	2.9
	Not use	78,392	77,704	71.5
Perception of distance from health center	Big problem	38,655	39,192	36.0
	Not big problem	69,506	69,532	64.0
Health insurance	Yes	10,763	10,944	10.1
	No	97,398	97,780	89.9
Cigarette smoke	Yes	1,003	984	0.91
	No	107,158	107,740	99.09
Residence	Urban	40,387	40,926	37.6
	Rural	67,774	67,798	62.4
Sub-Regions	Western	46,977	46,949	43.2
	Central	5,841	5,824	5.4
	Eastern	52,612	53,307	49.0
	Southern	2,731	2,644	2.4
Country level income	Low income	64,654	65,385	60.1
	Lower middle income	40,776	40,695	37.5
	Upper middle income	2,731	2,644	2.4

### Pooled prevalence of the co-occurrence of OWOB and anemia among reproductive-aged women in sub-Saharan Africa

The overall pooled prevalence of co-occurrence of overweight/obesity and anemia in reproductive women aged 15–49 was 12% (95% CI: 9–14%) with I^2^ of 99.7%, p-value<0.05. It covers a range of 3% in Burundi, Ethiopia and Rwanda to 26% in Mauritania (Fig 3). As shown from the forest plot, I^2^ was higher, indicating the variation across the 17 sub-Saharan countries was due to heterogeneity rather than chance. From these study participants with co-occurrence, 6,179 (52.1%) were obesity/overweight and mild anemia, 5,285 (44.6%) were obesity/overweight and moderate anemia, and 398(3.36%) were obesity/overweight and severe anemia.

Additionally, the pooled magnitude of OWOB and anemia was determined across country income levels. It was about 12% (95% CI: 9–14%) with I^2^ of 99.7%. the pooled estimate of overweight/ obesity and anemia was 9% (95%CI: 6–11%) in low-income countries, 17% (95% CI: 13–20%) in lower middle-income countries and 19% (95% CI: 17–20%) in an upper middle-income country. Since we have only one country per sub-region in Southern Africa and Central Africa, and only one country under upper middle-income countries, we cannot perform subgroup analysis to handle heterogeneity both at the regional and country-income levels.

**Fig 2:**
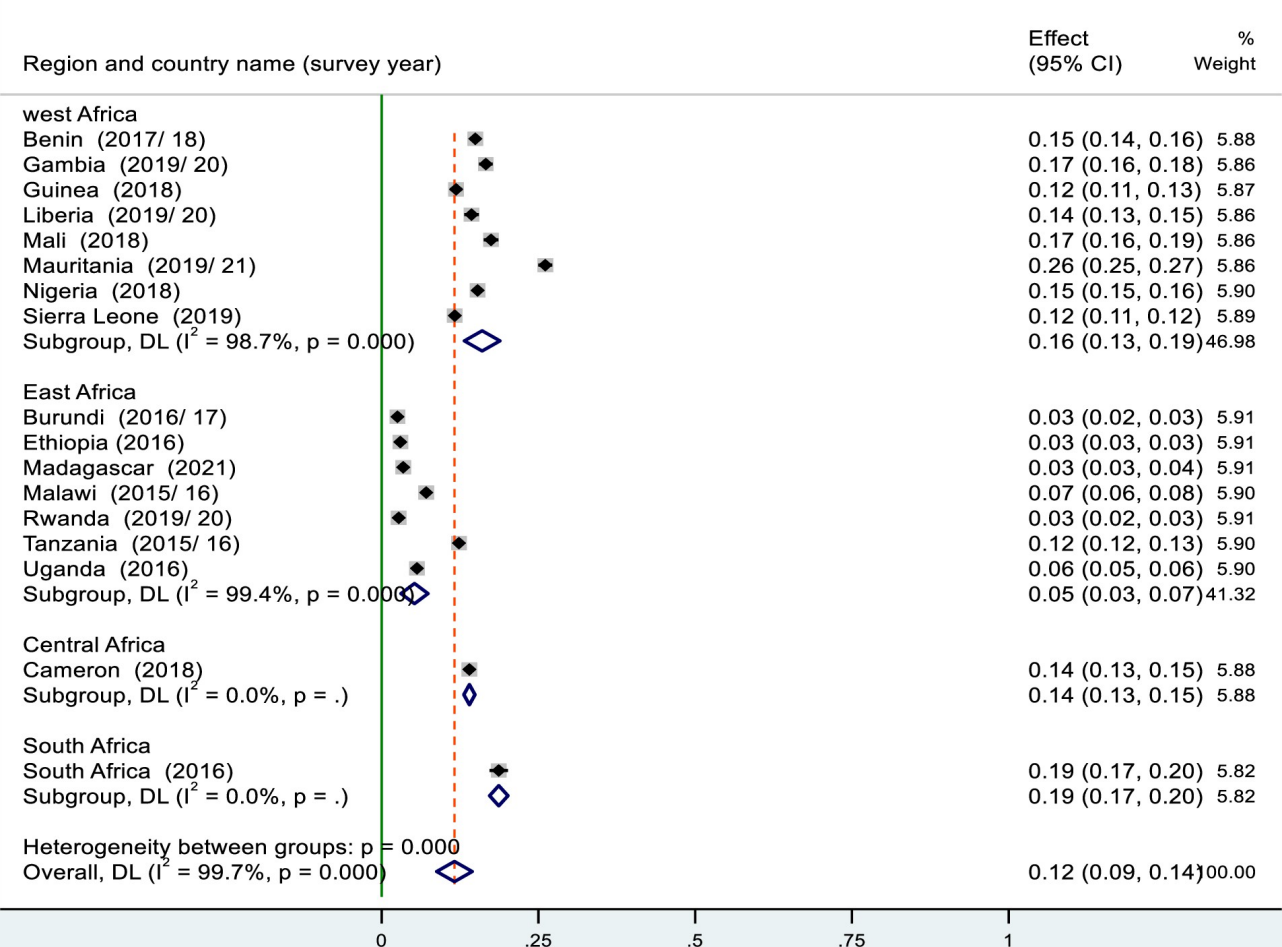
Forest plot showed the pooled prevalence of co-occurrence of OWOB and anemia among reproductive women 15–49 years in sub-Saharan Africa: DHS 2016–2021.

From the total weighted sample of 108,161, 29.41% were overweight/obese and the remaining 70.59% were non-overweight/obese. Depending on anemia status, 40.28% of women were anemic and from these anemic women, 20.05%, 18.67%, and 1.57%(1,694) were under the category of mild, moderate and severe anemia, respectively (Fig 2).

#### Spatial analysis of co-occurrence of overweight/obesity and Anemia among reproductive women in sub-Saharan Africa

**Fig 3:**
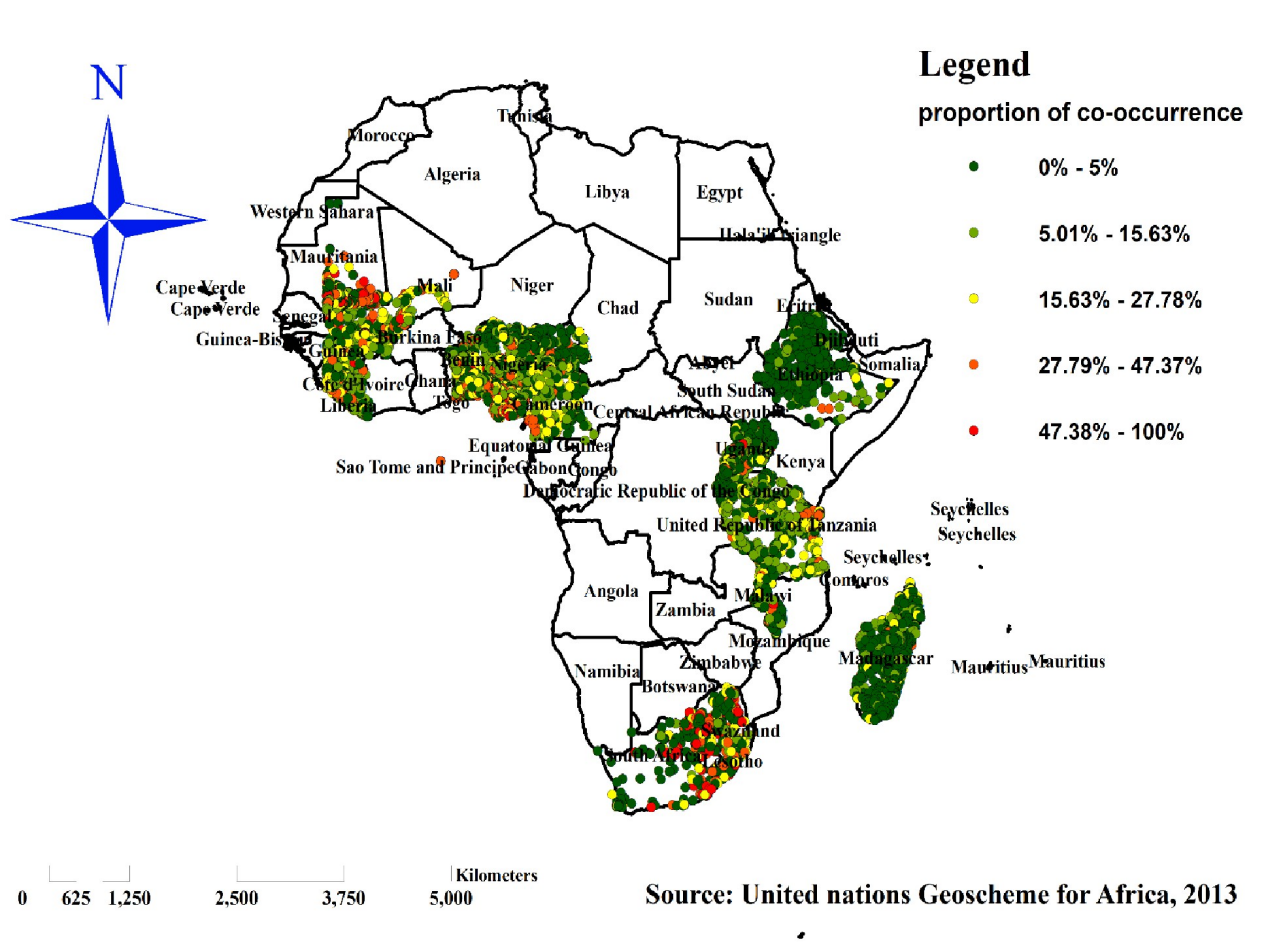
Spatial distribution of co-occurrence of overweight/obesity and anemia among reproductive women in sub-Saharan Africa, 2016–2021. Source: United Nations geoscheme for Africa, 2013.

Spatial distribution of co-occurrence of overweight/obesity and Anemia. The highest proportion of co-occurrence of overweight/obesity and anemia was spatially clustered in sub-Saharan Africa. In South Africa, Tanzania, Nigeria, Cameron, Mauritania, Mali and Guinea (Fig 3).

### Spatial autocorrelation analysis co-occurrence of OWOB and Anemia

**Fig 4:**
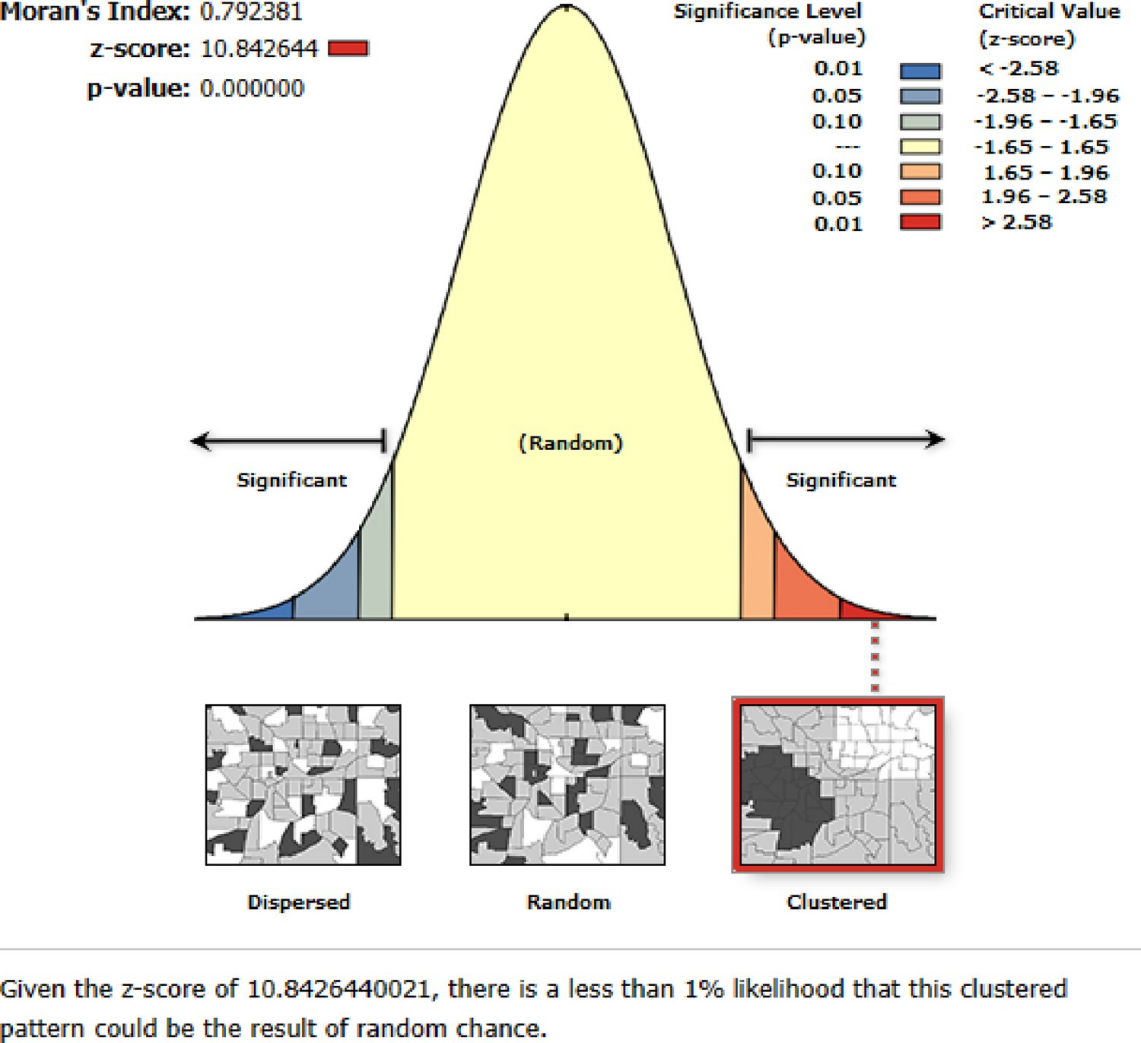
Spatial autocorrelation report of co-occurrence of overweight/obesity and anemia among reproductive women in sub-Saharan Africa, 2016–2021.

This study revealed that the spatial distribution of co-occurrence of overweight/obesity and anemia was found to be spatially clustered in Sub-Saharan Africa with Global Moran’s I 0.792381 (p < 0.001) (Fig 4).

### Incremental spatial autocorrelation co-occurrence of overweight/ obesity and anemia

**Fig 5:**
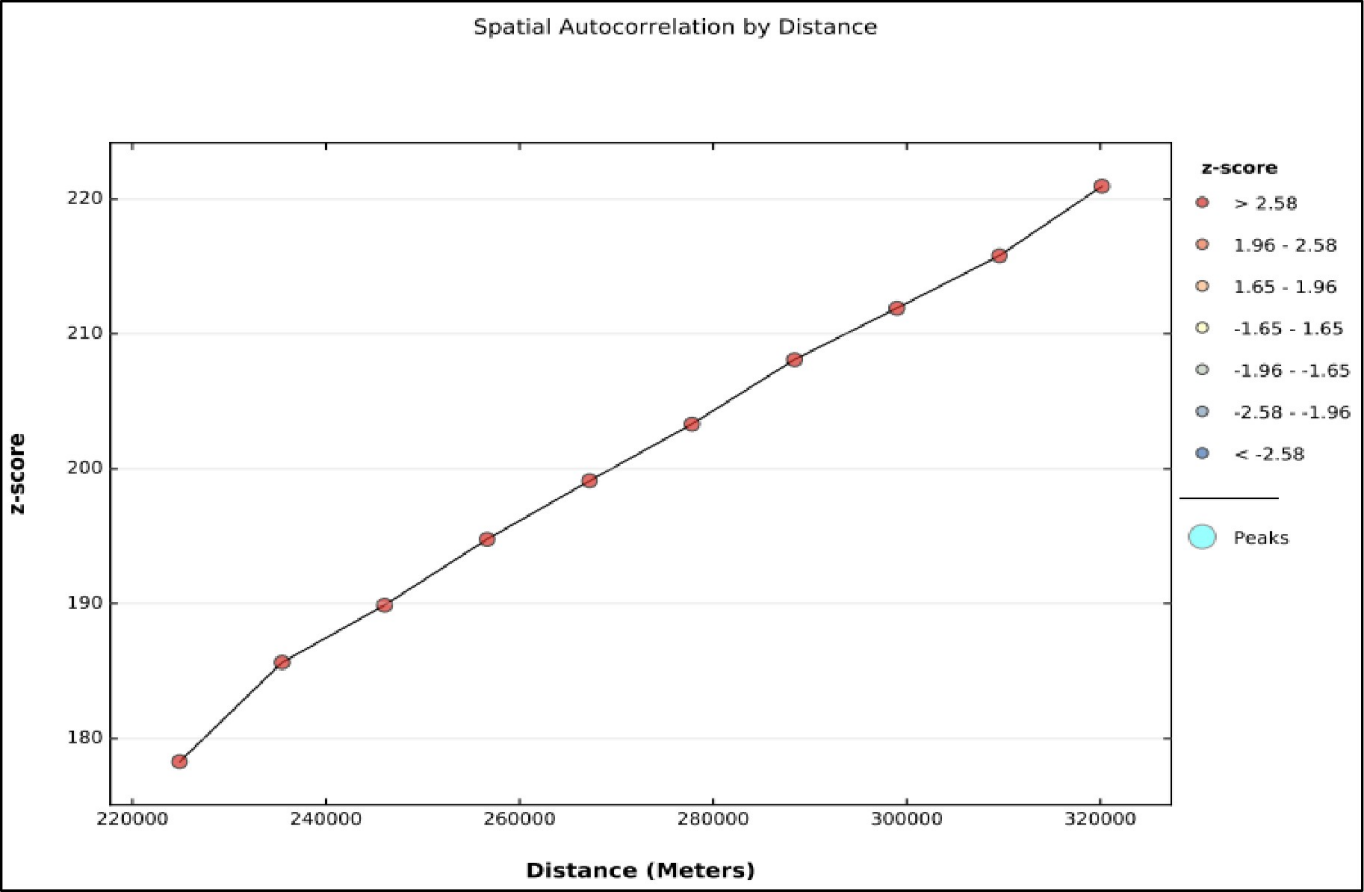
Incremental spatial autocorrelation report of co-occurrence of overweight/obesity and anemia among reproductive women in sub-Saharan Africa, 2016–2021.

Incremental spatial autocorrelation for a series of distance presented by a line graph with corresponding z-score was done to determine the average nearest neighbor and minimum and maximum distance band. A total of 10 distance bands were detected at a beginning distance of 224.886 Km with Z score 178.28 and no maximum peaks were observed. This is most often happens in cases where data has been aggregated and the scale of the processes impacting the input field variable is smaller than the aggregation scheme (Fig 5).

### Hotspot (Getis-Ord Gistatistic) analysis of co-occurrence OWOB and anemia

**Fig 6:**
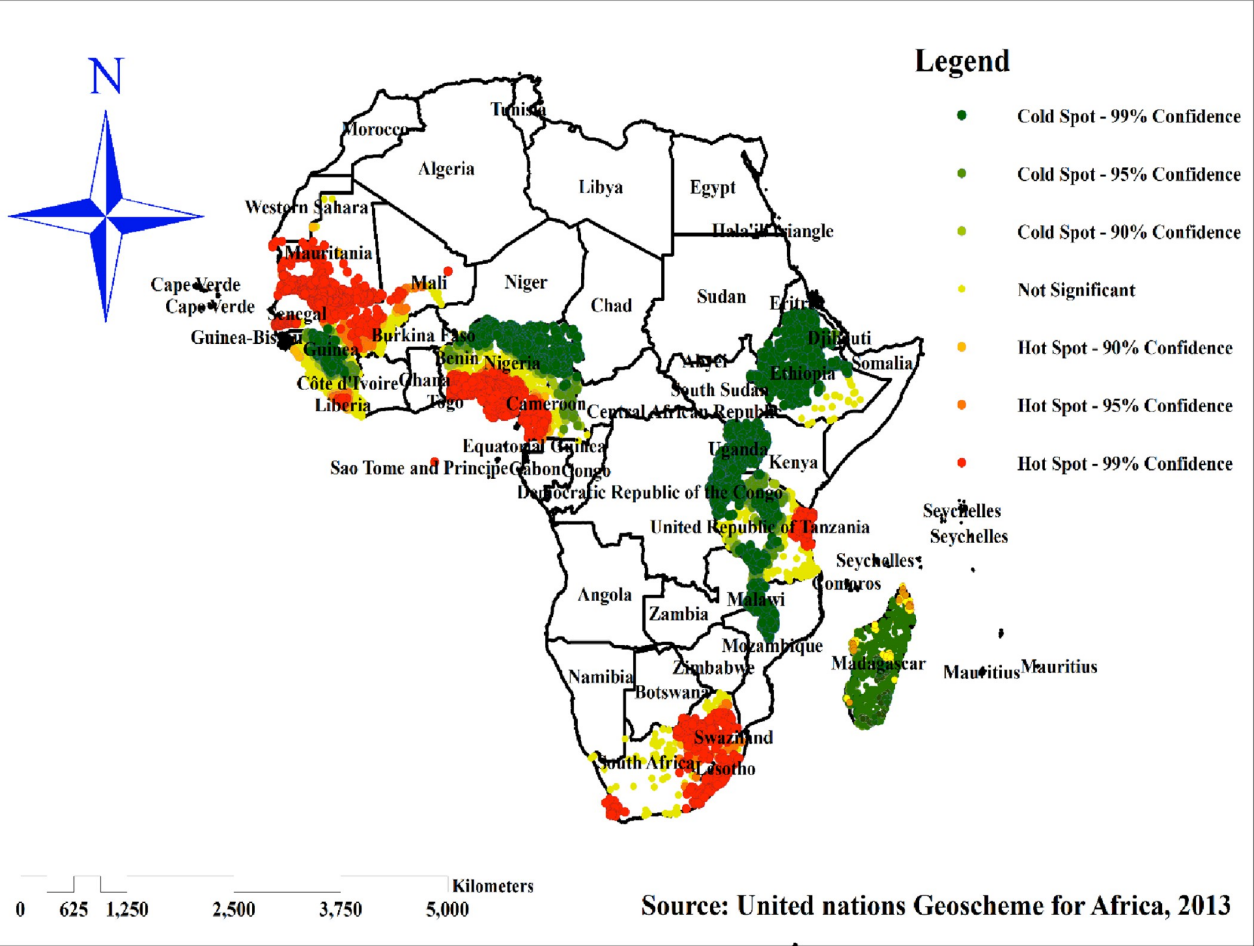
Hot-spot analysis of co-occurrence of overweight/obesity and anemia among reproductive women in sub-Saharan Africa, 2016–2021. Source: United Nations geoscheme for Africa, 2013.

Hotspot analysis was performed to identify areas with a high co-occurrence of overweight/ obesity and anemia among reproductive women in sub-Saharan Africa. The red color indicates significantly high co-occurrence areas and it is found in South Africa, Tanzania, Benin, Nigeria, Cameron, western Liberia, Mauritania and Mali, whereas the green color indicates areas with a low co-occurrence of overweight/obesity and anemia and is observed in Ethiopia, Uganda, Rwanda, Burundi, Madagascar, northern Guinea and northern Sierra Leone (Fig 6).

### Interpolation of co-occurrence of overweight/obesity and anemia

**Fig 7:**
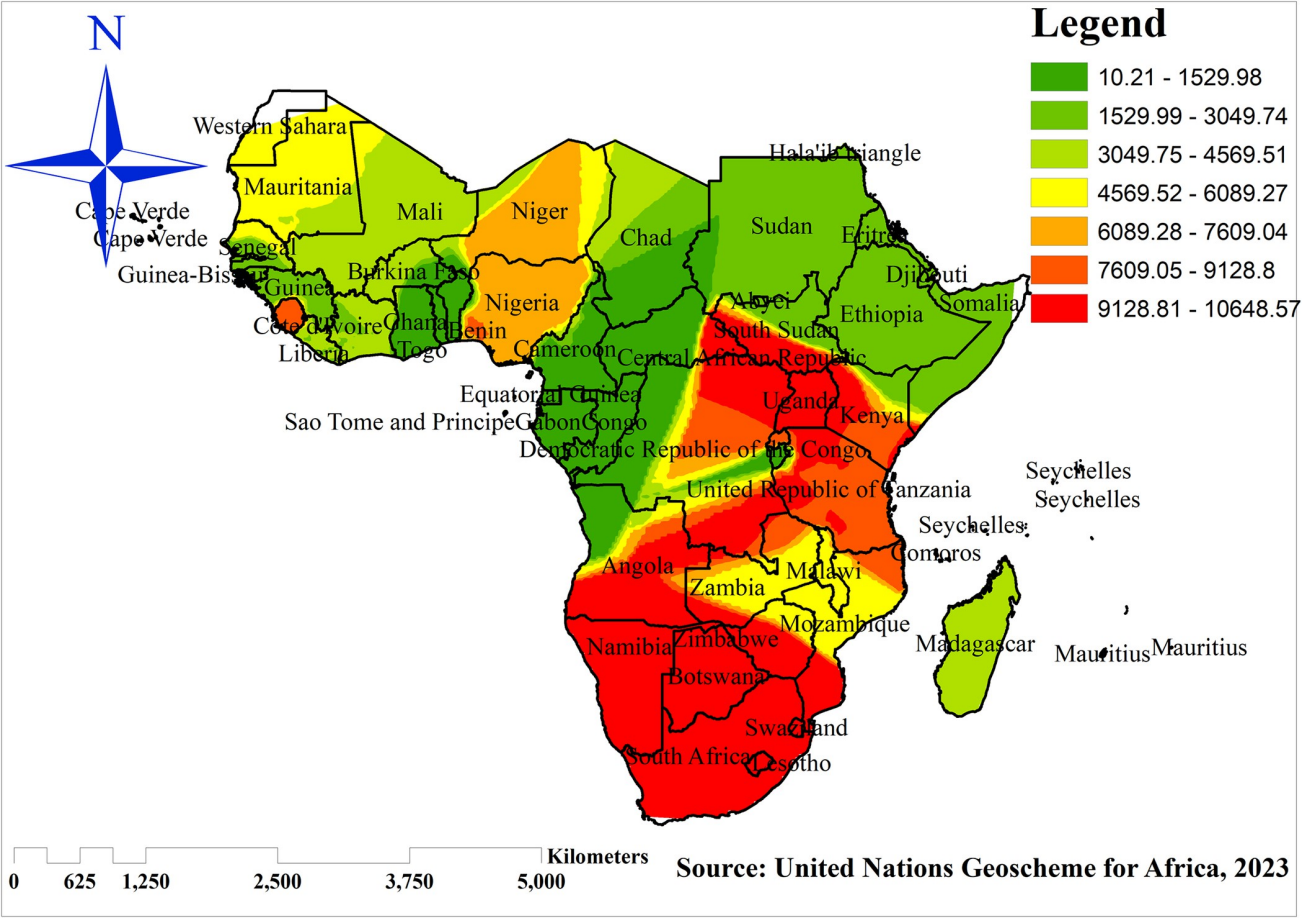
Spatial Kriging interpolation of co-occurrence of overweight/obesity and anemia among reproductive women in sub-Saharan Africa, 2016–2021. Source: United Nations geoscheme for Africa, 2013.

The map is made up of continuous images interpolated using the Kriging interpolation method, which predicts unknown values at other locations. The positive red color denoted a high likelihood of co-occurrence of overweight/obesity and anemia among reproductive women in sub-Saharan Africa. In contrast, the predicted low prevalence co-occurrence of overweight/obesity and anemia was identified by green color (Fig 7).

### Spatial SaTScan statistics analysis

The spatial scan statistics showed a total of 5,914 significant clusters, of which 5137 were most likely/primary, 154 were secondary and 623 were tertiary clusters. The most likely clusters were located in Liberia, Guinea, Gambia, Sira Leon, Mali, Cameron and Nigeria, which were centered at 4.964823 N, 7.656603 W) / 2160.73 km radius, Log-Likelihood Ratio (LRR) of 1687.30, Relative Risk (RR) of 2.58 at p-value < 0. 001, which implies that the women within spatial window have a 2.58 times higher likelihood of having co-occurrence of overweight/obesity and anemia as compared to women outside of the window. The secondary clusters’ spatial window was located in Tanzania, which was centered at 6.630457 S, 39.120270 E) with 198.36 km radius, Log-Likelihood Ratio (LRR) of 174.397, Relative risk (RR) of 2.05 at p-value 0. 001, which means that the women within the spatial window are 2.05 times more likely to develop co-occurrence of OWOB and anemia than women outside the window.

**Fig 8:**
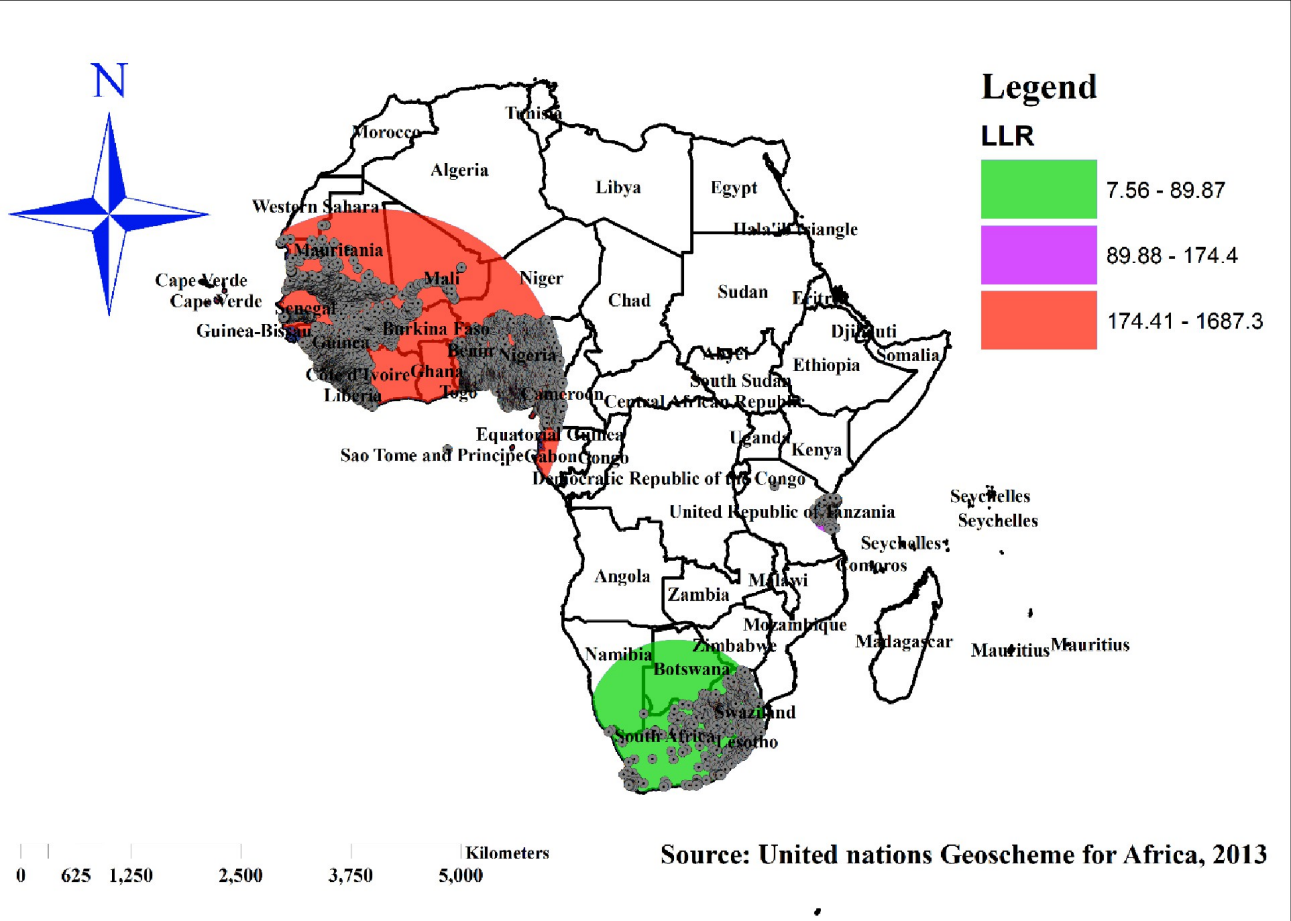
SaTScan analysis of co-occurrence of overweight/obesity and anemia among reproductive women in sub-Saharan Africa, 2016–2021. Source: United Nations geoscheme for Africa, 2013.

The tertiary clusters’ spatial window was located in South Africa that was centered at 27.185921 S, 23.324562 E with 883.66 km radius, Log-Likelihood Ratio (LRR) of 89.87, Relative risk (RR) of 1.82 at p-value 0. 001, which means that women within the spatial window have a 1.82 times higher likelihood of having co-occurrence of OWOB and anemia than women outside the window (Fig 8).

### Parameters and model comparison for multilevel analysis

The ICC in the model-one (null model) was 0.256, which indicates 25.6% of the variation in co-occurrence of overweight/obesity and anemia among reproductive women was attributed to cluster differences, while 74.4% were attributed to individual women factors.

The MOR value was 2.94 in the null model, indicating that the odds of co-occurrence of overweight/obesity and anemia among women were different across clusters.

Regarding the PCV value, 0.389 in the final model, points out that the 40% of the variation in co-occurrence of overweight/obesity and anemia among women was explained by the last model. In regard to the model fitness, deviance was checked and the model with the lowest deviance was the best model (Model-IV = 64176), selected for interpretation (Table 3).

**Table 3:** Parameters and model fit statistics for multilevel regression analysis models.

Parameters	Null model	Model II	Model III	Model VI
Cluster level variance	1.129	0.893	0.755	0.690
ICC	0.256*	0.214*	0.187*	0.173*
MOR	2.941	2.454	2.283	2.201
PCV	Reference	0.209	0.331	0.389
**Model fitness**				
Deviance	70,587.28	65,419.51	68,857.07	64,176.28
Mean VIF	-	1.07	1.44	1.48

*ICC*: Inter cluster correlation coefficient, *MOR*: Median odds ratio, *PCV*: Proportional Change in variance, *VIF*: Variance Inflation Factors.

*P-value < 0.05.

### Factors associated with co-occurrence of overweight/obesity and anemia among reproductive women

Both individual and community-level factors that have a p-value <0.2 in the bivariable analysis were selected for multilevel analysis. Based on the final model analysis of individual-level variables: age, marital status, educational status, occupation, media exposure, wealth index, type of toilet facility, contraceptive utilization, perception of distance from health facility and health insurance coverage were statistically significant variables associated with co-occurrence of OWOB and anemia, (p ≤ 0.05).

The odds of co-occurrence overweight/obesity and anemia were 1.91 (AOR = 1.91, 95%CI: 1.78, 2.04) among women aged 25–34 and 2.96 (AOR = 2.96, 95% CI: 2.76, 3.17) times higher among women aged 35–49 as compared to women aged 15–24 years. The odds of co-occurrence of overweight/obesity and anemia is 1.36 times (AOR = 1.36, 95% CI: 1.27, 1.46), 1.22 times (AOR = 1.22, 95%CI: 1.06, 1.40) and 1.36 times (AOR = 1.36 95% CI: 1.23, 1.50) higher among women who are married, widowed and divorced than single women, respectively. For women with primary education, the odds of co-occurrence of overweight/obesity and anemia was lower by 14% (AOR = 0.86, 95% CI: 0.81, 0.92) than women with no formal education. The odds of co-occurrence of overweight/obesity and anemia among women who have media exposure was 1.31 times (AOR = 1.31, 95%CI: 1.23, 1.39) higher than those who have no media exposure.

Higher odds of co-occurrence of OWOB and anemia exist among women of middle-income status had 1.19 times (AOR = 1.19, 95%CI: 1.11, 1.28) and rich had 1.36 times (AOR = 1.36, 95%CI: 1.26, 1.46) than poor individuals.

The odds of co-occurrence of OWOB and anemia for non-worker women was higher by 1.13 times (AOR = 1.13, 95% CI: 1.07, 1.19) as compared to the women who are working. The odds of co-occurrence of overweight/obesity and anemia was 1.17 times (AOR = 1.17, 95%CI: 1.10, 1.24) higher among women who had improved toilet facility than the counterpart.

The odds of co-occurrence of OWOB and anemia among women with traditional contraceptive utilization had 1.39 times (AOR = 1.39, 95%CI: 1.23, 1.58) and no contraceptive user 1.27 times (AOR = 1.27, 95CI: 1.20, 1.56) higher than women who use modern contraceptive methods.

Higher odds of co-occurrence of OWOB and anemia among women perceiving distance from the health facility as not a big problem as compared to women perceiving distance from the health facility as a big problem (AOR = 1.06: 95%CI: 1.01, 1.12). Reproductive-age women who had no health insurance coverage were 1.36 times (AOR = 1.36, 95%CI: 1.25, 1.49) higher co-occurrence of OWOB and anemia than women who had health insurance coverage.

From community-level variables place of residence and country income status were significantly associated with the co-occurrence of overweight/obesity and anemia. A woman who was living in an urban area had 1.61 times (AOR = 1.61, 95%CI: 1.50, 1.73) higher odds of co-occurrence of OWOB and anemia than a woman living in rural area. Higher odds of co-occurrence of OWOB and anemia among women 2.5 times (AOR = 2.50, 95% CI: 2.34, 2.66), 2.87 times (AOR = 2.87, 95% CI: 2.47, 3.34) in lower middle-income and upper middle-income countries, respectively than women from low-income countries (Table 4).

**Table 4:** Multilevel analysis of factors associated with co-occurrence of overweight/ obesity and anemia among reproductive women 15–49 years in sub-Saharan Africa: Based on 2016 to 2021 DHS.

Variable	Category	Null model	Model II	Model III	model IV
		AOR (95% CI)	AOR (95% CI)	AOR (95% CI)	AOR (95% CI)
Age	15–24 years	-	1	-	1
	25–34 years	-	2.06(1.92, 2.20)***	-	1.91(1.78, 2.04)***
	35–49 years	-	3.30(3.07, 3.53)***	-	2.96(2.76, 3.17)***
Marital status	Single	-	1	-	1
	Married	-	1.32(1.24, 1.42)***	-	1.36(1.27, 1.46)***
	Widowed	-	1.22(1.06, 1.40)**	-	1.22(1.06, 1.40)**
	Divorced	-	1.35(1.22, 1.49)***	-	1.36(1.23, 1.50)***
Educational attainment	No education	-	1	-	1
	Primary education	-	0.92(0.86, 0.98)**	-	0.86 (0.81,0.92)*
	Secondary & above	-	1.17(1.10, 1.25)***	-	0.99 (0.93,1.07)
Media exposure	Yes	-	1.42(1.33, 1.51)***	-	1.31(1.23, 1.39)***
	No	-	1	-	1
Wealth index	Poor	-	1	-	1
	Middle	-	1.26(1.17, 1.36)***	-	1.19(1.11, 1.28)***
	Rich	-	1.56(1.45, 1.67)***	-	1.36(1.26, 1.46)***
Occupation	Working	-	1	-	1
	Not working	-	1.21(1.15, 1.27)***	-	1.13(1.07,1.19)***
Source of water	Protected	-	1.06(1.00,1.13)*	-	1.05(0.99, 1.12)
	Unprotected	-	1	-	1
Type of toilet	Improved	-	1.18(1.12, 1.25)***	-	1.17(1.10, 1.24)***
	Unimproved	-	1	-	1
Contraceptive utilization	Modern	-	1	-	1
	Traditional	-	1.50(1.32, 1.68)***	-	1.39(1.23, 1.58)***
	Not use	-	1.34(1.27, 1.41)***	-	1.27(1.20, 1.56)***
Perception of distance from health center	Big problem	-	1	-	1
	Not big problem	-	1.12(1.06, 1.18)***	-	1.06(1.01, 1.12)**
Health insurance	Yes	-	1	-	1
	No	-	1.55(1.43, 1.70)***	-	1.36(1.25, 1.49)***
Residence	Urban	-	-	2.04(1.92, 2.16)***	1.61(1.50, 1.73)***
	Rural	-	-	1	1
Country income status	Low income	-	-	1	1
	Lower middle income	-	-	2.59(2.43, 2.76)***	2.50(2.34, 2.66)***
	Upper middle income	-	-	2.80(2.42, 3.23)***	2.87(2.47, 3.34)***

*AOR* Adjusted Odds Ratio, *CI* Confidence Interval.

Values expressed in Table 4.

*P-value < 0.05.

**P value < 0.01.

***P value < 0.001.

## Discussion

The co-occurrence of overweight/obesity and anemia is a global problem among reproductive women from 15–49 years. Identifying and reducing the preventable underlining factors is the critical mechanism to handle the consequences of the problem. Therefore, the current study aims to determine the spatial distribution and associated factors of co-occurrence of overweight/obesity and anemia among reproductive women in sub-Saharan Africa.

In our study, the pooled estimated magnitude of co-occurrence of overweight/obesity and anemia among reproductive women was 12% (95%CI: 9–14%), in our study. This finding is similar with a study done in Guatemala 12% and Brazil 14% [44], Low and middle income countries 12.4% [13]. It was higher than the finding in Ghana 7% [16], Malawi 3.4% [45], and lower than a study done in India 23.1% [46] and Philippines 23.7% [17]. The discrepancy across the world might be due to geographical variation, population growth, and socioeconomic status of the country, social, cultural and health variables [37,47]. Another possible reason is variation in food transition may have a potential linkage to the co-occurrence of overweight/obesity and anemia [48].

The spatial analysis found that the spatial distribution of co-occurrence of overweight/obesity and anemia across SSA countries was varied. Significant hotspot areas of co-occurrence of overweight/obesity and anemia were detected in South Africa, Tanzania, Benin, Nigeria, Cameron, western Liberia, Mauritania and Mali. This might be due to the inadequacy of maternal health care services related to nutrition and lack of awareness about the risk factors of overweight/obesity and anemia in those sub-Saharan countries. Besides, these countries are more on the transition of socioeconomic growth rapidly [16], and this finding suggests that women who have better economic status and are easily influenced by globalization were highly affected by the co-occurrence of OWOB and anemia. Therefore, public health programmers should design intervention strategies to reduce the co-occurrence of OWOB and anemia in these significant hotspot areas.

In this study, both individual and community-level variables have significant associations with the co-occurrence of overweight/obesity and anemia among reproductive women in SSA. The current study revealed that reproductive women aged 25–34 and 35–49 years were positively associated with co-occurrence of OWOB and anemia. A unit increase in age increases the odds of developing co-occurrence of overweight/obesity and anemia by 1.9 and 2.96 times higher for women aged 25–34 and 35–49 years respectively, than reproductive women from 15–24 years. This finding is consistent with a study done in Ghana and Philippines [16,17]. The possible reason might be due to biological changes increase in fat mass and a decreased basal metabolic rate contributes to overweight/obesity development as age increases [17]. There is evidence of metabolic interaction between being OWOB and anemic due to the demand for high blood volume required by the increase in body weight, inducing micronutrient deficiencies and a decrease in the bioaccessibility of iron due to its sequestration in the reticuloendothelial system [49].

In line with the existing literature [16], our study indicated that women who were Married, divorced and widowed had the 1.36, 1.22 and 1.36 times higher odds of co-occurrence of overweight/obesity and anemia than single women. This is due to the fact that being married and transition in marital status either divorced or widowed increase in body weight than unmarried women [50]. Married women gain weight during pregnancy and sustained lifetime if weight loss not occurred in the postpartum period and increase in substantial demand for iron [51]. Additionally, an increase in parity and use of hormonal contraceptives may lead to the co-occurrence of OWOB and anemia among married, widowed and divorced women than single (unmarried) women [51,52].

This study indicated that reproductive women with a primary level of education were negatively associated with co-occurrence of OWOB and anemia compared to women with no education. Even though, there are some literatures [17,53], stated that women who attain a higher level of education were more likely to develop co-occurrence of overweight/obesity and anemia, our finding revealed that the odds of co-occurrence of OWOB and anemia among women with a primary level of education was lower by 14% than non-educated. This might be because reproductive women who are educated are expected to get more information from different sources about appropriate dietary intake and health care services regarding the co-occurrence of overweight/obesity and anemia [54]. Moreover, non-educated women are mostly less likely to seek health care treatment like anemia or OWOB which result in poor maternal health, and hence have an increased risk of weight gain and the simultaneous existence of anemia [55,56].

The result of our study points out those reproductive women who had media exposure was positively associated with the co-occurrence of OWOB and anemia. This was supported by a study in India [56,57]. The possible reason might be reproductive women who have media access, had good economic growth and limited lifestyle modification or transition of feeding habits to an energy-dense and nutrient-poor diet resulting in the development of co-occurrence. Another reason might be women who are watching television or using internet for a long time adopt a sedentary lifestyle, reducing physical activity and increasing the risk of overweight/obesity. Overweight/obesity causes metabolic disturbance among reproductive women and ends up with anemia [58].

Likewise, the wealth index was associated with co-occurrence of OWOB and anemia. As observed from our findings, women who were in the middle and rich wealth quantile had higher odds of developing co-occurrence of overweight/obesity and anemia. Similarly, findings from Ghana [16], Malawi [18] and low and middle-income countries [59], revealed that reproductive women in the middle and richest wealth index were highly affected by co-occurrence of OWOB and anemia. This could be due to a shift from traditional foods to diverse diets containing energy-dense and nutrient-poor foods that are the main contributors of overweight/obesity and micronutrient deficiency among most socioeconomically advantaged individuals [53,60]. Again, overweight/obesity can co-occur regardless of dietary iron intake. Empirical evidence from different kinds of literature shows that overweight/obesity is characterized by chronic low-grade inflammation (elevated IL-6), increases hepcidin (an iron-regulating peptide hormone), reduces intestinal iron absorption, and eventually leads to systemic iron deficiency and/or iron restricted erythropoiesis [61,62].

We found that reproductive women who are not working had higher odds of co-occurrence of overweight/obesity and anemia. This result is congruent with a study in India and Myanmar showing that non-working women have higher odds of developing co-occurrence of overweight/obesity and anemia than workers [63]. The possible justification might be a reduction in the level of physical activity that induces sedentary life and progression to the development of OWOB and anemia, in contrast with the earlier lifestyle [64].

Consistent with a previous study in India [65], reproductive women who were on traditional contraceptives and did not use any contraceptive methods have higher odds of developing co-occurrence of overweight/obesity and anemia than those women who used modern contraceptive methods. The reason might be women who were using modern contraceptives for a long time are linked to weight gain due to hormonal effects. Therefore, the occurrence of overweight/obesity is mainly due to the pharmacodynamic effect of both progesterone and estrogen. In fact, progesterone increases appetite and results in faster metabolism, while estrogen facilitates lipid metabolism and fat accumulation in the adipose tissue in the cell [22]. On the contrary, using those modern contraceptive methods; especially hormonal contraceptives had a high potential to improve the hemoglobin level among women of childbearing age [66]. Because of this paradoxical effect among modern contraceptive users, the co-occurrence of overweight/obesity and anemia is less likely to develop than for non-users and those on traditional methods.

Compatible with previous literature [56,67], the result of our study shows that women who have access to improved type of toilet have higher odds of developing the co-occurrence of overweight/obesity and anemia. This might be due to the fact that individuals who have good socioeconomic status have improved toilet facility and it prevents human contact with human excreta and physically closer sanitation facility. On the contrary, reproductive women with poor sanitation facility might be exposed to communicable diseases which will have a considerable impact on their nutritional status [24].

In this study, we found that women who consider distance from health facility as not a big problem has a positive association with co-occurrence of overweight/obesity and anemia. This indicates that women with co-occurrence of OWOB and anemia were found near the health services and in the most advantageous socioeconomic group. The possible reason might be that women who are found near health care services may not utilize maternal health services effectively like iron folate supplementation, modern contraceptive use, and a continuum of care despite overweight/obese.

Accordingly, reproductive women who have no health insurance coverage were significantly associated with co-occurrence of overweight/obesity and anemia. Even though no study has been done before regarding our topic, this might be due to the fact that having health insurance coverage encourages reproductive women to consult weight control mechanisms [68], and in the counterpart, a study in Mali demographic survey implies that childbearing age women who have health insurance coverage more likely to be anemic than those not covered by health insurance [69]. The possible reason for this discrepancy might be the government in Mali’s health insurance does not cover cases of anemia because of poor quality of service delivery [70]. But for our study, mothers who have no health insurance coverage face financial barriers to access health care services and develop a higher co-occurrence of overweight/obesity and anemia than those who have health insurance coverage.

In the current study, women from urban areas had higher odds of developing co-occurrence of overweight/obesity and anemia than those from rural areas. Similarly, a study conducted in Malawi [18], India, Myanmar and Nepal [63], and Bangladesh [71], revealed that co-occurrence of overweight/obesity and anemia is highly associated with urbanization. This is due to the fact that exposure to an urban environment may induce a nutritional transition and the adoption of western lifestyle [72]. While rich women can afford to access expensive junk foods that have added fat, salt, and sugar content, while poor women are forced to consume cheap diets with limited nutrients [73].

Moreover, the factor that has a potential association with the co-occurrence of overweight/obesity and anemia was country income status. The finding from this study indicates, reproductive women who are living in lower middle-income and upper middle-income countries of SSA, have higher odds of developing co-occurrence of overweight/obesity and anemia than those women in low-income countries. This is likely due to the fact that increment in wealth status parallel with fast socioeconomic growth in middle-income countries results in greater access to processed food, an escape from physical labor and joining a sedentary lifestyle, and consumption of food with poor dietary quality, which could contribute to a higher risk of overweight/obesity and anemia co-occurrence [74].

### Strength and limitation of study

The strength of this study was the use of spatial analysis with a larger sample to detect significant hotspot areas of co-occurrence of overweight/obesity and anemia to plan location-specific and effective intervention programs. Another strength was the use of DHS data after sustainable development goal endorsement, which gives recent information on the co-occurrence of OWOB and anemia. However, this study has some limitations. The first, cross sectional nature of DHS data, the study could not show cause-effect relationships. Second, SaTScan analysis detects only circular clusters. Therefore, irregularly shaped clusters may be missed. Third, the Kriging interpolation method takes the claim that the space under study is stable and that the joint probability does not change across the study area. As a result, the interpolated values in non-stationary areas might be higher or lower than the true values. The GPS (latitude and longitude) data were taken from the enumeration area, which is displaced to 5km in urban areas and 10 km in rural areas for privacy issues and this might bias our spatial distribution.

## Conclusion

The magnitude of co-occurrence of overweight/obesity and anemia among reproductive women aged 15–49 years in sub-Saharan Africa was relatively high. In sub-Saharan Africa, co-occurrence of overweight/obesity and anemia had spatial variation across the sub-regions. Significant hotspot areas of co-occurrence of overweight/obesity and anemia were found in South Africa, Tanzania, Benin, Nigeria, Cameron, western Liberia, Mauritania and Mali whereas the cold spot areas of co-occurrence were observed in Ethiopia, Uganda, Rwanda, Burundi, Madagascar, Guinea and northern Sierra Leone.

Individual level variables such as age above 25 years, being married, widowed and divorced, not working, having media exposure, middle and rich wealth status, no contraceptive utilization and being a user of traditional contraceptive methods, improved toilet facility, perception of distance from health facility not a big problem and no health insurance coverage have a positive association, whereas the primary level of education has a negative association with co-occurrence of overweight/obesity and anemia among reproductive women. From community-level variables urban residency, and being from lower and upper middle-income countries were statistically significant associations with co-occurrence of overweight/obesity and anemia.

Moreover, to decrease the co-occurrence of overweight/obesity and anemia among reproductive women in SSA, we recommend public health programmers and other stalk holders to work together with other sectors and focus on those hotspot areas of co-occurrence.

### Sub-Saharan Africa countries Ministry of health

It is recommended to design plans that can give responses for different forms of malnutrition mainly overweight/obesity and anemia, and health care service access equity to reproductive women for improving early awareness about their health status prior to pregnancy.

There should be expanded screening programs to detect the coexistence of these conditions in women of childbearing age and/or at least prior to conception.

In order to move forward, evidence-based strategies which focus on either prevention or treatment of the co-occurrence of overweight/obesity and anemia should be implemented.

### Sub-Saharan Africa countries Ministry of education

They should involve in many intervention activities such as delivering information to encourage healthy nutritional habits, physical activity and taking iron supplementation.

Establish promotion strategies in early childhood, starting from exclusive breastfeeding and timely initiation of complementary feeding practice to the life course, so that girls become healthy adult women free of malnutrition.

### For the government of Sub-Saharan Africa countries

The national government should be responsible for implementing the Sustainable Development Goals as a way to improve the economy, health care systems, food security and gender equality.

It is also recommended to use social safety nets such as food transfers, subsidies, and/or vouchers as necessary per context that discourage intake of high-energy, low-nutrient foods and give rewards for the consumption of nutritious foods.

Another recommendation forwarded to the national government is to scale up antenatal care that includes counseling about healthy eating and keeping physically active before pregnancy to stay healthy and prevent excessive weight gain, along with micronutrient utilization.

The implementation of policies aimed at transforming food systems to ensure all have the option of healthier diets and environments for feeding.

### For researchers

Researchers coming in this area should give high emphasis to reproductive women who are living in these countries under areas of hotspots.

Furthermore, a nationally representative primary study with better study design is recommended to detect cause-effect relationship of co-occurrence of overweight/obesity and anemia among reproductive women.

## Supporting information

S1 Data Appended data. (DTA)

## Abbreviations

BMI: Body Mass Index
DHS: Demographic Health Survey
EA: Enumeration Areas
GDHS: Ghana Demographic Health Survey
Hb: Hemoglobin
ICC: Intraclass Correlation Coefficient
LLR: Log-Likelihood Ratio
LR: Likelihood Ratio
NPW: Non-Pregnant Women
OWOB: Overweight or Obesity
SDG: Sustainable Development Goals
SSA: Sub-Saharan Africa
TDHS: Tanzania Demographic Health Survey
UNICEF: United Nations International Children’s Emergency Fund
WHO: World Health Organization
